# Automated grading and diagnosis of sacroiliitis on CT images using a 3D convolutional neural network: a multicenter retrospective study

**DOI:** 10.1038/s41598-026-44911-9

**Published:** 2026-03-18

**Authors:** Yong-ku Du, Run Liu, Hua Guo, Chao Li, Pei Chen, Hang Qiu, Dan-dan Shi, Jie Cheng, Jun Yan, Yi-shan Li

**Affiliations:** 1 Department of Radiology, Xi’an Fifth Hospital, Xi’an, China; 2 Department of Radiology, Xi’an Center Hospital, Xi’an, China; 3 Xi’an Key Laboratory of Metabolic Disease Imaging, Xi’an No.3 Hospital, Affiliated Hospital of Northwest University, Xi’an, 710000 China; 4 Department of Rheumatology, Xi’an Fifth Hospital, Xi’an, China

**Keywords:** Ankylosing spondylitis, Sacroiliitis, 3D convolutional neural network, Computed tomography, Computational biology and bioinformatics, Diseases, Health care, Mathematics and computing, Medical research

## Abstract

Radiographic grading of the sacroiliac joints plays a critical role in the differential diagnosis of ankylosing spondylitis (AS) and in guiding the treatment. The aim of this study was to develop an automated 3D convolutional neural network (3D CNN) for grading and diagnosing sacroiliitis on CT images to assist clinicians. This study included CT images from 2,144 participants, comprising healthy controls and patients with suspected ankylosing spondylitis (AS). A V-Net based segmentation model was applied, followed by training a three-dimensional DenseNet-based convolutional neural network (3D CNN) for both five-class and three-class classification tasks. Grading by three radiologists according to the New York criteria served as the reference standard. The model’s diagnostic performance was evaluated on an external multicenter validation set and compared with radiologist interpretations. For the five-class task, the model’s area under the receiver operating characteristic curve (AUC) for grades 0–IV were 0.966, 0.937, 0.881, 0.962, and 0.994, respectively. In the simplified three-class task, AUCs for classes 0, 1, and 2 were 0.984, 0.967, and 0.994, respectively. On the external validation set, three-class AUCs were 0.957, 0.934, and 0.992. With AI assistance, two radiologists’ diagnostic accuracy improved by 6.9% and 8.4%, respectively. The proposed segmentation–classification framework enables accurate and reproducible CT grading of sacroiliitis.

## Introduction

Ankylosing spondylitis (AS) is a chronic inflammatory disease that primarily affects the axial skeleton and sacroiliac joints^1,2^. Clinical diagnosis depends largely on radiographic grading of the sacroiliac joints, which is critical for differential diagnosis and treatment planning^3^. According to the modified New York criteria, definite sacroiliitis is defined as bilateral sacroiliitis grade ≥ II or unilateral sacroiliitis grade III–IV. However, sacroiliitis grading is subjective and prone to inter-observer variability, even among experienced radiologists and rheumatologists^4,5^. Increasing imaging volumes further burden radiologists, and prolonged, labor-intensive interpretation may increase the risk of diagnostic error or oversight.

Compared with radiography, computed tomography (CT) provides superior spatial resolution and enables more accurate assessment of structural changes in the sacroiliac joints, such as erosions and ankylosis. In settings without access to subspecialist radiologists, or when MRI is unavailable, CT-based diagnostic tools could reduce missed diagnoses during opportunistic screening.

Artificial intelligence (AI) has been widely applied across healthcare, including risk stratification and medical image analysis, improving diagnostic efficiency and accuracy^6^. Therefore, this study aims to develop an automated CT-based evaluation system using a three-dimensional convolutional neural network (3D CNN) to assist clinicians in diagnosing sacroiliitis and to mitigate the subjectivity, inefficiency, and inconsistency of conventional assessment.

## Materials and methods

**Fig. 1:**
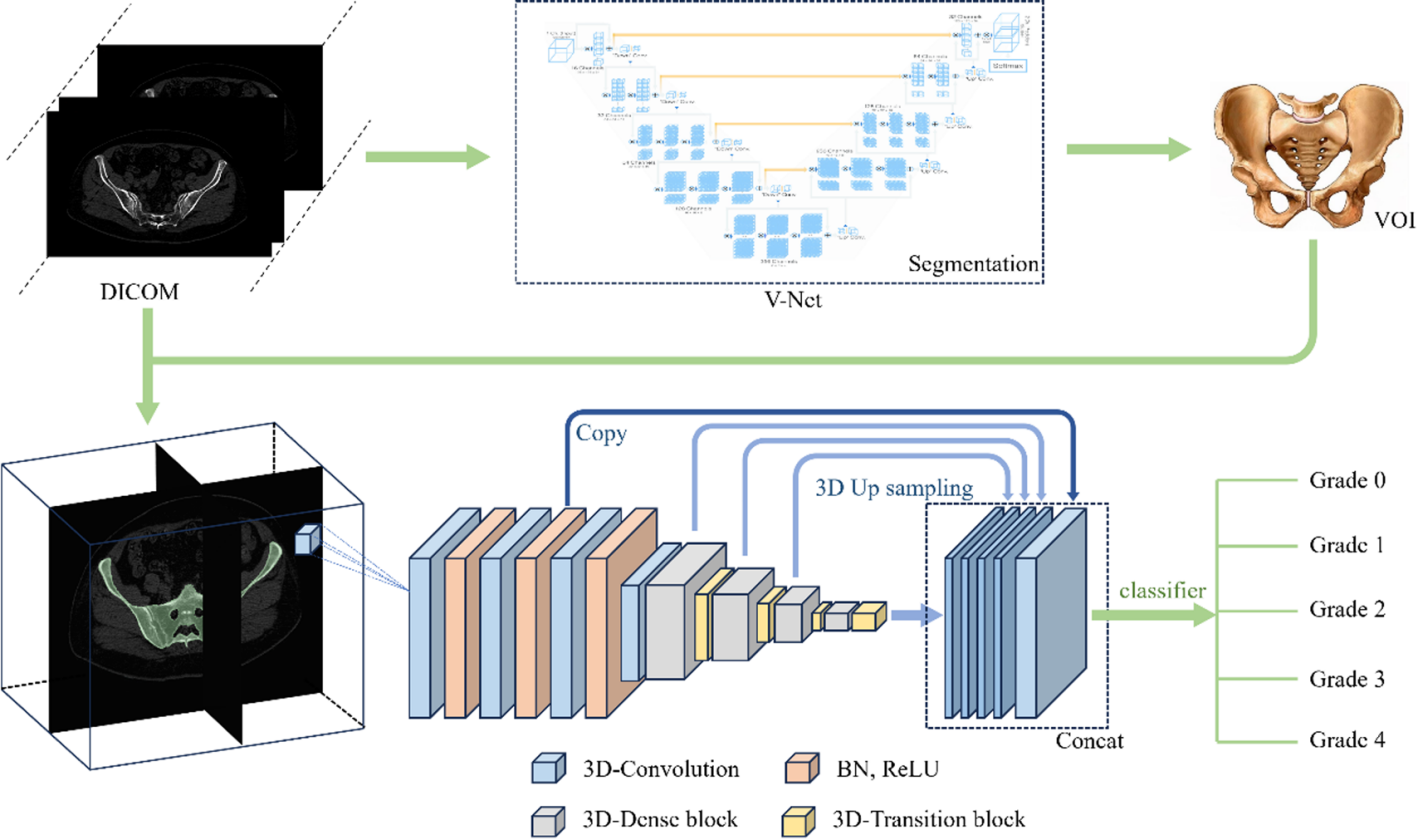
Flowchart of the automatic evaluation system for sacroiliitis based on three-dimensional convolutional neural network.

The workflow of the proposed 3D CNN–based automated sacroiliitis evaluation system is shown in Fig. 1. CT images from 2,144 subjects were included. Grading by three radiologists according to the modified New York criteria^3^ served as the reference standard for model development. All images were segmented using a V-Net–based segmentation network to extract the pelvic region of interest (ROI). The segmented ROIs and corresponding labels were used to train a three-dimensional DenseNet–based classification network for five-class and three-class grading. The trained model performs end-to-end automated grading of sacroiliac joint CT images. Its diagnostic performance was evaluated on an internal test set, an external multicenter validation set, and in human–AI collaboration experiments.

### Study population

**Fig. 2:**
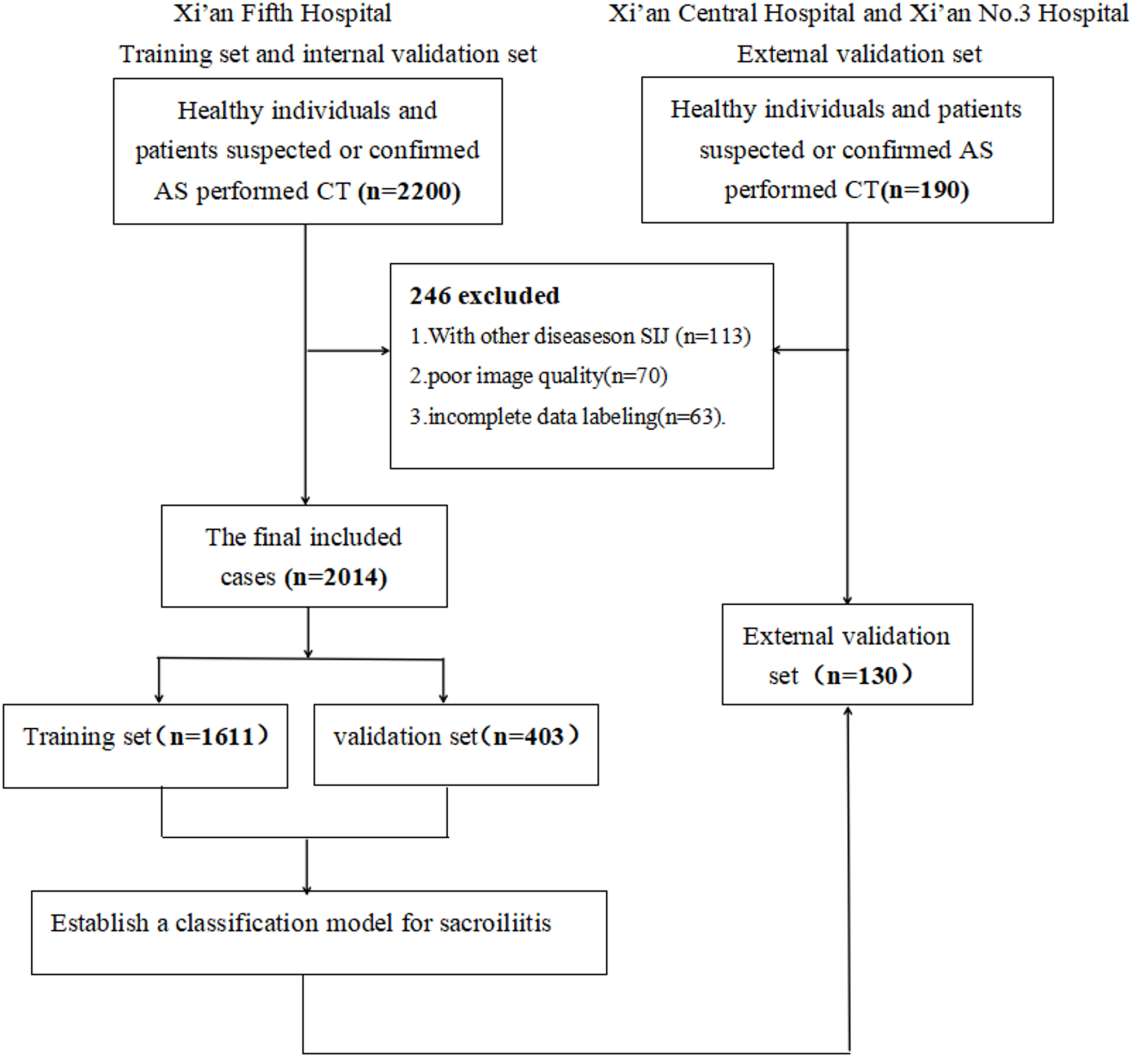
Flowcharts of the training set, internal validation set and external validation.

A total of 2,480 CT scans from healthy individuals and patients with suspected ankylosing spondylitis (AS) were retrospectively collected between March 2019 and April 2024 (2,200 from Xi’an Fifth Hospital, 95 from Xi’an No.3 Hospital, and 94 from Xi’an Central Hospital). After screening, we excluded 113 cases of condensing osteitis and infectious sacroiliitis, 70 scans with severe artifacts or poor image quality, and 63 cases with incomplete annotation, resulting in 2,144 subjects included in the analysis. To ensure adequate class representation during training and to assess generalizability, data from Xi’an Fifth Hospital were split into training (*n* = 1,611) and internal test (*n* = 403) sets at an 8:2 ratio. Data from Xi’an No.3 Hospital and Xi’an Central Hospital (*n* = 130) were used as the external test set. The study flow is shown in Fig. 2.

Inclusion criteria: (1) healthy individuals or patients suspected of AS; (2) age 20–70 years; (3) complete clinical and laboratory data. Exclusion criteria: (1) condensing osteitis, infectious sacroiliitis, or primary/secondary tumors of the sacroiliac joints; (2) severe artifacts or poor image quality precluding diagnosis; (3) incomplete annotation.

CT scans for the training and internal test sets were acquired on a GE Optima 680. External validation scans were acquired on multiple scanners, including Philips iCT 256, Philips Spectral 256, United Imaging Healthcare (UIH) uCT 768, Siemens SOMATOM go.Now, and Siemens SOMATOM Definition. Original DICOM images were retrieved from each site’s PACS. The study was registered in the Chinese Clinical Trial Registry (ChiCTR2500096632) and approved by the Ethics Committee of Xi’an Fifth Hospital (Approval No. 2024-84), the requirement for informed consent was waived and patient identifiers were anonymized.

### CT grading of sacroiliitis

**Fig. 3:**
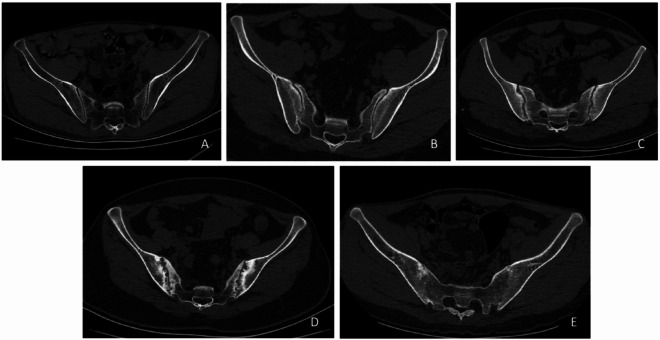
(**A**–**E**) illustrates representative examples: (**A**) Grade 0; (**B**) Grade 1; (**C**) Grade 2; (**D**) Grade 3; (**E**) Grade 4.

The 1984 revised American College of Rheumatology (New York) criteria (in Table 1) are the standard for grading structural damage on sacroiliitis radiographs. Because plain radiographs have limited diagnostic sensitivity for sacroiliitis, CT-based grading in clinical practice commonly follows these radiographic criteria, as in previous studies^7–9^. In this study, CT images were independently graded by two radiologists (each with > 10 years’ experience) using a five-point scale (Grades 0–4): Grade 0, normal; Grade 1, suspected abnormality with blurring and irregularity of the sacroiliac joint surfaces; Grade 2, mild sclerosis or erosion of the articular surfaces without joint-space change; Grade 3, joint-space narrowing with partial ankylosis (fusion); Grade 4, loss of joint space with extensive or complete ankylosis. Discrepancies were resolved by a senior musculoskeletal radiologist, and a consensus grade was assigned, AS shown in Fig. 3.

**Table 1:** Graded by trained and calibrated readers using the modified New York criteria.

Grades	Radiographic assessment
Grade 0	Normal
Grade 1	Suspicious abnormality: limited bone erosion is only seen in a single layer
Grade 2	Minimal abnormality: mild sacroiliac joint sclerosis and erosion, without observable changes in joint space
Grade 3	Unequivocal abnormality: narrowing of the sacroiliac joint space, accompanied by partial articular surface fusion
Grade 4	Severe abnormality: the joint space complete ankylosis, most or all tetanus, joint fusion

### Image segmentation

To obtain standardized 3D inputs, sacroiliac joint CT volumes were automatically segmented to isolate the pelvic region using a V-Net–based convolutional neural network from the United Imaging Intelligence (uAI) Research Portal. The model’s encoder–decoder architecture with skip connections provided end-to-end semantic segmentation of pelvic bony structures, producing binary 3D masks. Each pelvic mask was used to crop the region of interest from the original DICOM volumes; the cropped data were then reconstructed and normalized into 3D image patches that served as input to the subsequent 3D CNN for sacroiliitis grading.

### Data preprocessing and model training

This study employed a Densely Connected Convolutional Network (DenseNet) to extract 3D spatially discriminative features from sacroiliac joint CT images. In DenseNet, each layer concatenates feature maps from all preceding layers, which promotes feature reuse, improves gradient flow, mitigates vanishing gradients, and enhances multi-scale feature fusion. The network comprises multiple dense blocks linked by transition layers that use 1 × 1 × 1 convolutions and pooling to compress channels and reduce feature-map dimensions, thereby controlling overfitting and improving generalization. High-dimensional features were aggregated globally and passed to fully connected layers, with a Softmax output producing a probability distribution over five severity grades. Compared with architectures such as AlexNet and ResNet, DenseNet has demonstrated superior performance in 3D medical image classification because its dense connectivity enables more efficient feature reuse and more stable gradient propagation while preserving richer original information via feature concatenation, making it well suited for sacroiliitis grading.

Model training was performed on an Ubuntu 18.04 workstation with two NVIDIA GPUs. The implementation used Python 3.7 and the fastai API built on PyTorch. Preprocessing included resampling CT volumes to an isotropic resolution of 2.0 mm and cropping fixed-size ROIs of 128 × 128 × 128 voxels from the segmented pelvic region. Class-balanced sampling ensured uniform class representation each epoch. Online data augmentation included random center offsets (random center cropping = [5, 5, 5]). Images were normalized with a fixed normalizer (mean = 400, SD = 650). The network was trained with the Adam optimizer (β1 = 0.9, β2 = 0.999), an initial learning rate of 1 × 10 − 4, and a step-wise scheduler that reduced the learning rate by a factor of 0.1 after 1000 epochs. Training ran for 1000 epochs with a batch size of 4.

### Statistical analysis

Statistical analyses were performed using SPSS version 21.0 and Python 3.7. Categorical variables were presented as counts (n) and percentages (%), and continuous variables with a normal distribution were presented as mean ± standard deviation. Model performance was evaluated using receiver operating characteristic (ROC) curves and confusion matrices. The area under the ROC curve (AUC) with 95% confidence intervals (CIs), sensitivity, specificity, positive predictive value (PPV), negative predictive value (NPV), and overall accuracy were calculated. A P-value < 0.05 was considered statistically significant.

## Results

### Patient characteristics

This study included 2144 sacroiliac joint CT scans from multiple centers, divided into a training set (*n* = 1611), an internal test set (*n* = 403), and an external validation set (*n* = 130). The mean patient age was 40.5 ± 12.5 years; 1222 were male (57.0%) and 922 were female (43.0%). According to the grading standard, grades 0–IV comprised 308 (14.4%), 531 (24.8%), 384 (17.9%), 564 (26.3%), and 357 (16.6%) cases, respectively. Detailed baseline clinical characteristics and grade distribution are presented in Table 2.

**Table 2:** The number of images used for train and test.

Grade	Train	Test	External test	Total
Age	40.4 ± 12.3	40.4 ± 13.1	41.4 ± 12.8	40.5 ± 12.5
Sex				
Male	924	229	69	1222
Female	687	174	61	922
Grading				
Grade 0	225	56	27	308
Grade I	410	102	19	531
Grade II	277	70	37	384
Grade III	421	105	38	564
Grade IV	278	70	9	357
All	1611	403	130	2144

### Accuracy verification of the segmentation-classification model

To assess the accuracy and feasibility of the two-stage design, we compared its diagnostic performance with that of an end-to-end approach (i.e., feeding raw CT images directly into the classifier). As shown in Table 3, the end-to-end approach achieved an accuracy of 0.533, whereas the two-stage pipeline (V-Net segmentation followed by classification) achieved an accuracy of 0.744. Incorporating V-Net segmentation produced an absolute accuracy increase of 0.211, corresponding to a relative improvement of 21.1% over the end-to-end model.

To quantitatively evaluate the V-Net segmentation module, 40 samples were randomly selected from the test set and manually annotated by a senior radiologist. The model achieved a mean Dice Similarity Coefficient (DSC) of 0.95 ± 0.02 and a mean Intersection over Union (IoU) of 0.91 ± 0.03, indicating a strong overlap between the segmentation results and the gold-standard annotations. This high concordance ensures accurate extraction of regions of interest (ROI) for downstream classification tasks.

**Table 3:** Comparison of different input strategies and 3D DenseNet for five-class sacroiliitis grading.

Model	Params (M)	FLOPs (G)	Accuracy	AUC	Specificity
3D BasicNet	0.6	5.5	0.623 (0.578–0.667)	0.903 (0.885–0.919)	0.906 (0.895–0.917)
3D ResNet-50	46.5	82.0	0.643 (0.598–0.690)	0.902 (0.884–0.919)	0.911 (0.900-0.922)
3D DenseNet for End-to-End	11.2	28.6	0.533 (0.486–0.586)	0.853 (0.829–0.878)	0.883 (0.872–0.896)
3D DenseNet	11.2	28.6	0.744 (0.700-0.787)	0.954 (0.942–0.964)	0.936 (0.925–0.947)

### 3D performance evaluation of 3D DenseNet for sacroiliitis grading

To address class imbalance, we applied balanced sampling. For each grade (0–IV), we calculated accuracy (ACC), sensitivity (recall), specificity, F1 score, and area under the receiver operating characteristic curve (AUC). 95% confidence intervals were estimated using 1,000 bootstrap iterations. Macro-, micro-, and weighted-average metrics are reported in Table 4.

**Table 4:** Performance metrics of the 3D-DenseNet for 5-class grading.

Category	AUC	Sensitivity (recall)	Specificity	Precision	F1-Score
Grade 0	0.966 (0.949–0.981)	0.786 (0.680–0.889)	0.954 (0.932–0.974)	0.733 (0.618–0.844)	0.759 (0.660–0.843)
Grade 1	0.937 (0.912–0.958)	0.667 (0.571–0.759)	0.930 (0.899–0.958)	0.764 (0.674–0.849)	0.712 (0.632–0.781)
Grade 2	0.881 (0.842–0.915)	0.471 (0.343–0.587)	0.916 (0.883–0.945)	0.541 (0.416–0.656)	0.504 (0.383–0.595)
Grade 3	0.962 (0.943–0.978)	0.857 (0.784–0.922)	0.896 (0.859–0.929)	0.744 (0.664–0.820)	0.796 (0.735–0.850)
Grade 4	0.994 (0.988–0.999)	0.929 (0.859–0.986)	0.979 (0.961–0.994)	0.903 (0.825–0.970)	0.915 (0.862–0.963)
Macro avg	0.948 (0.936–0.960)	0.742 (0.700-0.782)	0.935 (0.924–0.946)	0.737 (0.695–0.779)	0.737 (0.694–0.777)
Weighted avg	0.948 (0.934–0.960)	0.744 (0.700-0.787)	0.931 (0.917–0.944)	0.740 (0.697–0.786)	0.740 (0.695–0.784)
Micro average	0.954 (0.942–0.964)	0.744 (0.700-0.787)	0.936 (0.925–0.947)	0.744 (0.700-0.787)	0.744 (0.700-0.787)

**Fig. 4:**
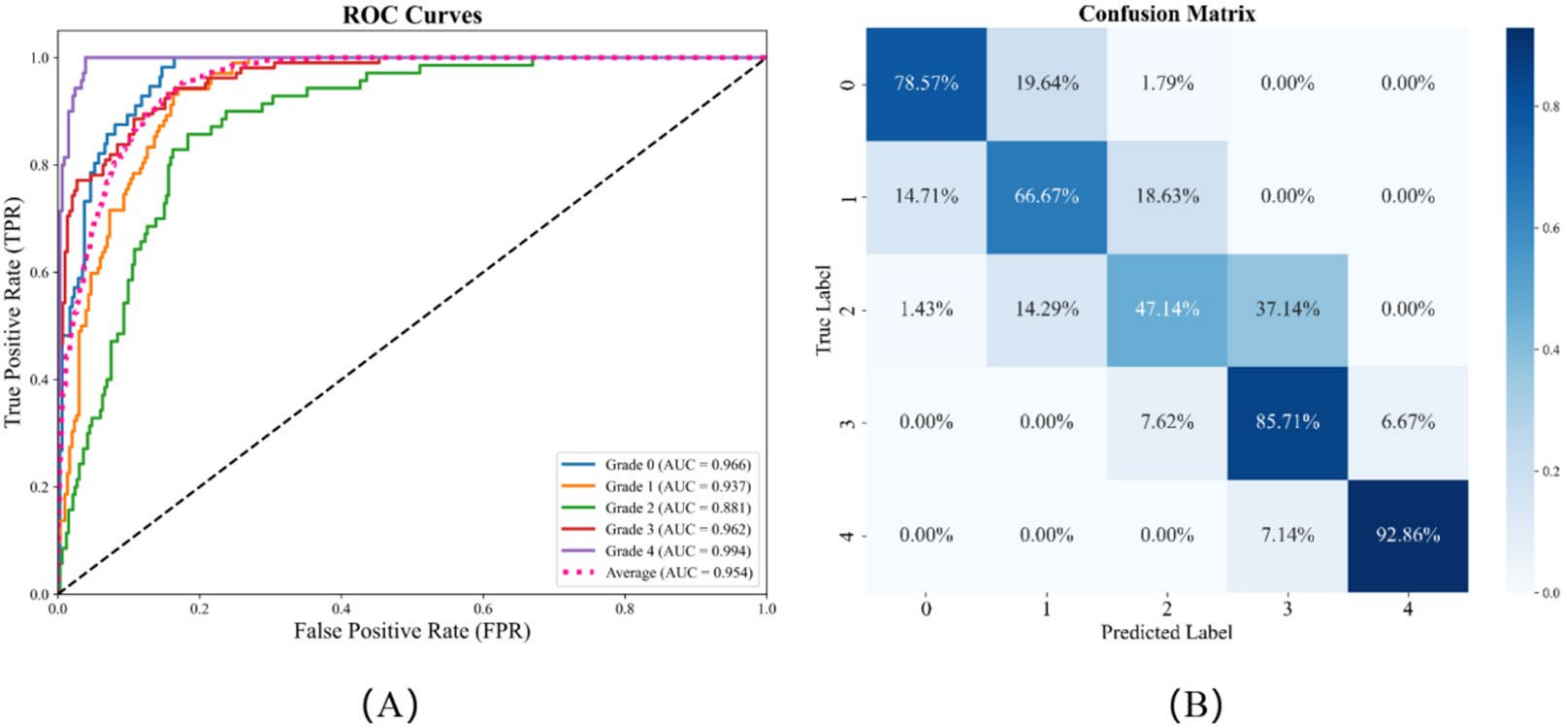
Diagnostic performance of the five-class grading model: (**A**) ROC curves for Grades 0ཞ4; (**B**) Confusion matrix.

In the five-class task (grades 0–IV), the proposed 3D DenseNet demonstrated strong diagnostic performance across grades. ROC analysis yielded AUCs of 0.966, 0.937, 0.881, 0.962, and 0.994 for grades 0–4, respectively, with a mean AUC of 0.954 (Fig. 4A). Grade 4 achieved the highest AUC (0.994), indicating excellent identification of advanced sacroiliitis; grade 2 had the lowest AUC (0.881), suggesting greater difficulty distinguishing early-to-mid stages.

The confusion matrix (Fig. 4B) corroborated these findings: accuracy was highest for grades 0 and 4 (44 and 65 correctly classified cases, respectively), and grade 3 also showed strong recognition (90 correct). Misclassifications were concentrated between grades 1 and 2, reflecting overlapping imaging features in early disease. Overall, the five-class model supports refined grading for stratified diagnosis and progression monitoring.

**Fig. 5:**
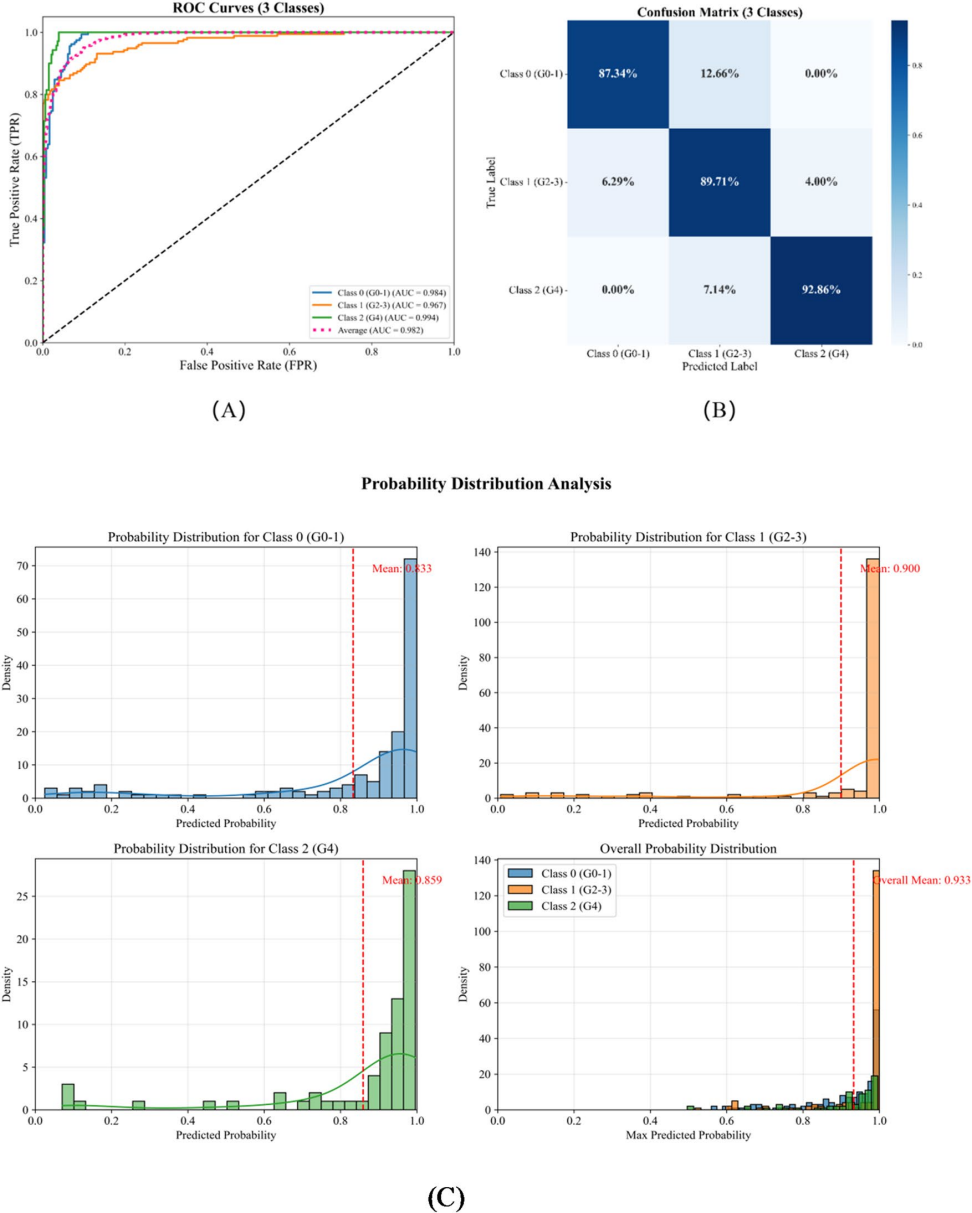
Diagnostic performance of the three-class classification model: (**A**) ROC curves; (**B**) Confusion matrix; (**C**) Probability distribution analysis.

For greater clinical utility, we collapsed the original five-grade scoring system into three actionable categories: Category 0 (normal/suspicious): sacroiliitis grades 0–I; Category 1 (structural damage): grades II–III; Category 2 (ankylosis): grade IV. This grouping follows the modified New York criteria, which require at least grade Ⅱ sacroiliitis for a definitive ankylosing spondylitis (AS) diagnosis. ROC-curve analysis (Fig. 5A) yielded AUCs of 0.984, 0.967, and 0.994 for categories 0, 1, and 2, respectively, with a mean AUC of 0.982 (5% CI: 0.973–0.988). The model’s performance metrics were: sensitivity 0.893, specificity 0.931, positive predictive value 0.895, negative predictive value 0.931, and overall accuracy 0.893. Advanced lesions (category 2) were identified most accurately, whereas discrimination was slightly lower for the intermediate stage (category 1). Although merging grade Ⅰ into the negative category improves diagnostic specificity and reduces over-diagnosis, it lowers sensitivity for detecting very early lesions. Because CT primarily visualizes chronic structural damage, early inflammatory changes (preceding structural alteration) are better assessed clinically with MRI.

The confusion matrix (Fig. 5B) shows 138/158 (87.3%) Class 0 and 65/70 (92.9%) Class 2 cases were correctly identified; Class 1 had 157/175 correct classifications, with most errors into adjacent classes, consistent with transitional imaging features.

Probability distribution analysis (Fig. 5C) indicated high model confidence: average predicted probabilities for Classes 0–2 were 0.933, 0.900, and 0.859, respectively, and most predictions exceeded 0.8, indicating stable classification. In summary, while the five-class system provides finer granularity, the three-class framework demonstrated greater stability and reliability for clinical application, particularly in distinguishing early (grades 0–1) from advanced (grade 4) disease.

**Fig. 6:**
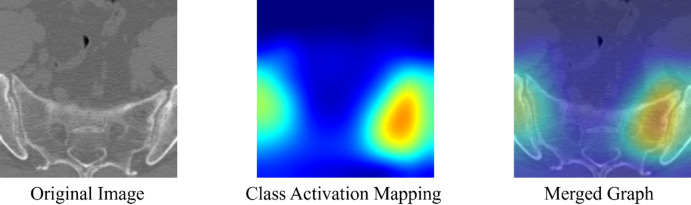
Grad-CAM visualization: The first Image shows the original CT; the second Image shows the Class Activation Map; the third Image shows the Fused Image (Red regions indicate high model attention/activation).

Figure 6 presents original CT images, class activation maps, and fused overlays. These visualizations show that the model focused on the sacroiliac joints rather than irrelevant structures, supporting clinical interpretability.

### External validation of 3D DenseNet

**Fig. 7:**
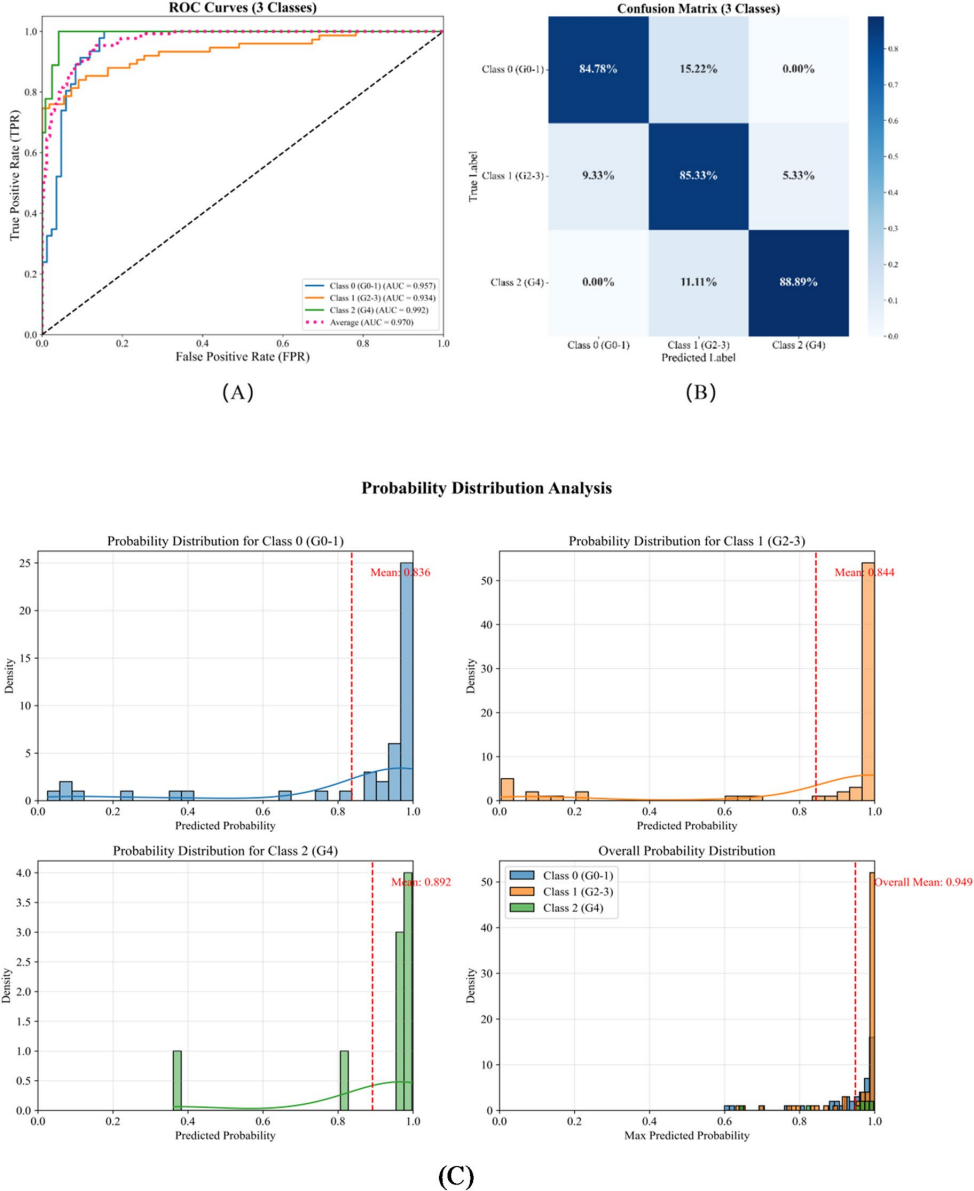
External validation of the three-class classification model: (**A**) ROC curves; (**B**) confusion matrix; (**C**) probability distribution analysis.

In the external validation cohort, the three-class model maintained strong diagnostic performance. ROC analysis (Fig. 7A) yielded AUCs of 0.957, 0.934, and 0.992 for Classes 0–2, respectively, with a mean AUC of 0.970 (95% CI: 0.950–0.986). Overall sensitivity, specificity, positive predictive value (PPV), negative predictive value (NPV), and accuracy were 0.854, 0.884, 0.859, 0.861, and 0.854, respectively, indicating excellent discrimination across classes.

The confusion matrix (Fig. 7B) shows the model correctly identified 39 Class 0, 64 Class 1, and 8 Class 2 cases. Errors were primarily between adjacent classes (e.g., 0 vs. 1, 1 vs. 2), consistent with the progressive nature of sacroiliitis and overlapping imaging features across stages.

Probability distribution analysis (Fig. 7C) reported average predicted probabilities of 0.836, 0.944, and 0.892 for Classes 0–2, respectively; the overall average predicted probability was 0.949. These results support model stability on an independent dataset and indicate high confidence and reliability for clinical application.

### Comparison of 3D DenseNet with radiologist interpretation

**Fig. 8:**
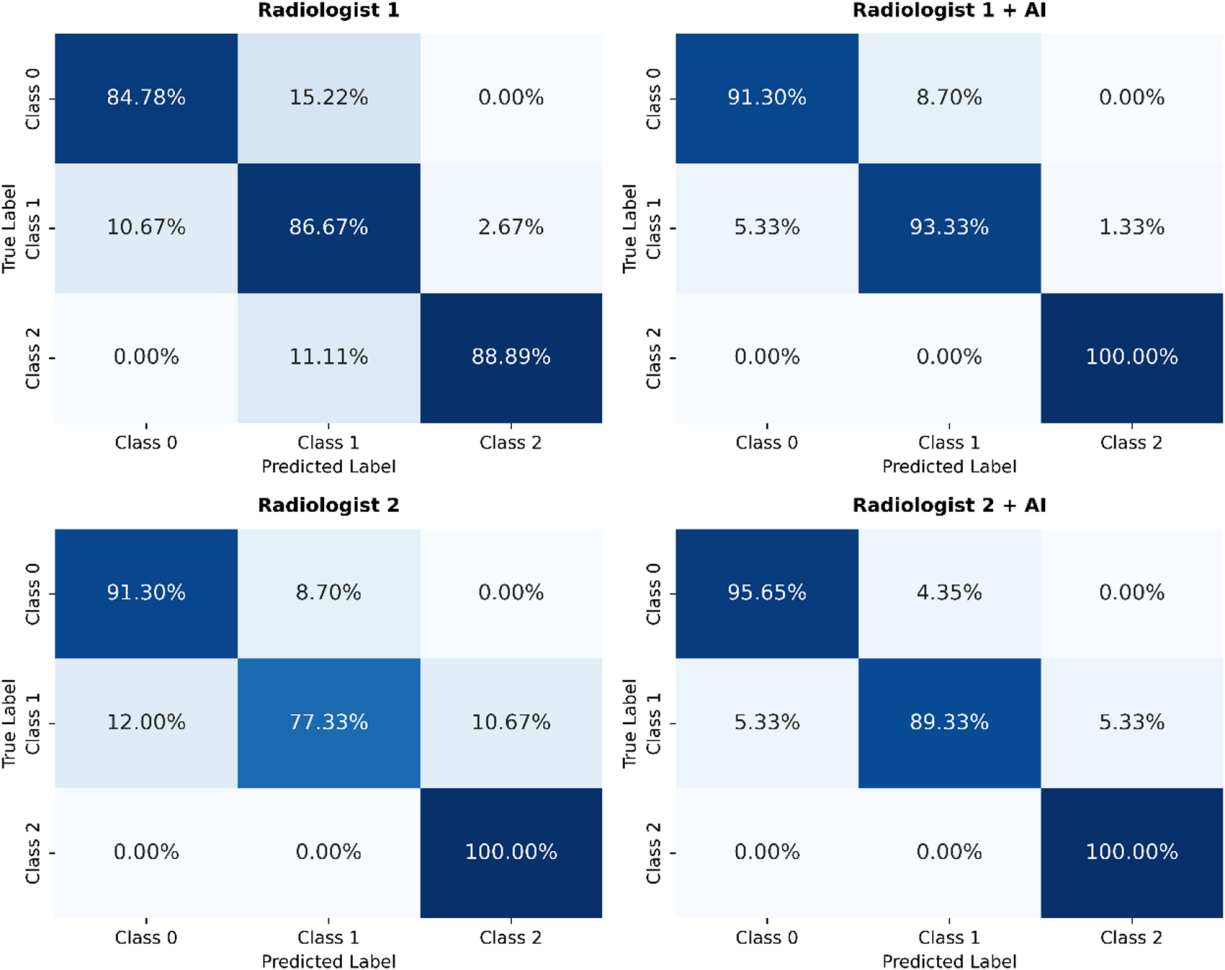
Confusion matrices of radiologists with and without AI assistance.

To assess observer variability and clinical utility, we conducted a human–AI collaboration study on the external validation set. Two radiologists (R1 and R2) first graded cases independently and then regraded them with AI assistance. With AI support, agreement scores increased for R1 and R2 from 0.745 to 0.720 to 0.872 and 0.862, respectively. AI assistance produced absolute improvements in diagnostic accuracy of 6.9 and 8.4% points for R1 and R2, respectively. As shown in Table 5; Fig. 8.

**Table 5:** Impact of AI assistance on radiologists’ diagnostic performance for sacroiliitis grading on the external validation set (three-class task).

Method	Accuracy	Cohen’s Kappa	Sensitivity	Specificity	Precision
AI Model	0.854	0.734	0.854	0.884	0.859
Radiologist 1	0.862	0.745	0.868	0.914	0.853
Radiologist 1 + AI	0.931	0.872	0.949	0.957	0.933
Improvement	+ 6.9%	+ 0.127			
Radiologist 2	0.839	0.720	0.896	0.918	0.802
Radiologist 2 + AI	0.923	0.862	0.950	0.961	0.895
Improvement	+ 8.4%	+ 0.142			

## Discussion

Ankylosing spondylitis (AS) is a chronic inflammatory disease. Early detection of structural lesions allows prompt treatment, prevents irreversible joint damage, improves patients’ quality of life, and reduces societal costs^10^.

Pelvic radiographs are often used for initial screening and diagnosis of AS because of their simplicity^11^, but many studies report low reliability for detecting sacroiliitis^12,13^. Magnetic resonance imaging (MRI) has higher diagnostic value and can identify active sacroiliitis at an earlier stage^2,14^; however, its high cost, contraindications, and long scan time limit its use as a primary screening tool^15^. In resource-limited settings where MRI is unavailable, computed tomography (CT) remains the reference standard for structural bone lesions and plays an important role in diagnosing spondyloarthritis^16,17^.

Recent years have seen rapid development and widespread application of artificial intelligence (AI), machine learning and other digital tools to support clinical decision-making and reduce manual workload^18,19^. Three-dimensional convolutional neural networks (3D-CNNs), a subset of deep learning, have achieved strong performance on medical tasks, sometimes matching or surpassing human experts in areas such as lung cancer detection and classification^20^, brain tumor classification^21^, gastric cancer prognosis prediction^22^, and bioinformatics^23,24^. Several studies have applied CNNs to sacroiliitis, but these reports often had small sample sizes^25–27^, focused on binary classification (for example, erosion/ankylosis detection or AS/non-AS classification)^28–30^, or lacked external validation^31,32^.

In this study, we developed a 3D DenseNet model using a large CT dataset from the rheumatology department of Xi’an Fifth Hospital, and validated it with external multicenter data and a human–AI collaboration study. Unlike prior binary-class studies, we implemented a five-grade classification system to better reflect disease progression. The model achieved area under the ROC curve (AUC) values greater than 0.95 for grades 0, III, and IV, indicating strong discrimination between normal joints and advanced sacroiliitis. Lower AUCs for grades I and II are consistent with clinical reality, because imaging changes overlap between adjacent grades and inter-observer variability exists even among radiologists. Overall, the 3D DenseNet demonstrated high diagnostic performance for the five-class task.

To improve clinical applicability, we also trained a three-class model by grouping the five grades into clinically meaningful categories: Grades 0–I as Class 0, Grades II–III as Class 1, and Grade IV as Class 2. For the internal test set, the three-class model yielded AUCs of 0.984, 0.967, and 0.994 for Classes 0, 1, and 2, respectively (average AUC 0.982). External validation produced AUCs of 0.957, 0.934, and 0.992 (average AUC 0.970), indicating robust discriminatory ability. The model produced well-separated probability distributions, supporting interpretable and confident predictions. Performance was best for advanced lesions (Class 2) and slightly lower for the intermediate stage (Class 1), likely due to feature overlap and reader variability between adjacent grades.

We further evaluated the model’s assistive value in a human–AI collaboration study. With AI assistance, inter-observer agreement improved markedly (R1 + AI: 0.872; R2 + AI: 0.862 vs. R1: 0.745; R2: 0.720). AI assistance increased the two radiologists’ diagnostic accuracy by 6.9% and 8.4%, respectively, demonstrating that AI-assisted CT grading can improve reproducibility and reduce the subjectivity of visual interpretation.

Compared with a prior study^7^, our dataset was approximately five times larger (2144 vs. 435 cases) and drawn from multiple centers, enabling complete five-grade classification rather than a coarse three-class system. Our 3D DenseNet al.so achieved higher overall accuracy (0.854 vs. 0.802). The human–AI collaboration study quantified the model’s clinical utility, showing about an 8% improvement in radiologists’ diagnostic accuracy.

Clinically, this system offers several advantages. It provides objective, reproducible assessment of sacroiliac joint changes, reducing inter-observer variability. The hierarchical design (five-class and three-class outputs) supports both clinical triage and disease monitoring. External validation across multiple scanner models showed high performance, suggesting compatibility and practical utility across vendors. Finally, probability outputs from the deep learning model enhance diagnostic confidence and may facilitate clinician adoption. In summary, the developed 3D DenseNet showed excellent discriminative ability across classes and stable performance across populations and imaging conditions, supporting its potential for clinical implementation.

Although promising results were obtained in this study, several limitations remain. First, this was a retrospective study; although it involved multiple centers, all subjects were recruited from the same region, which may introduce selection bias. Second, class imbalance existed in the external validation set, especially the limited sample size of grade Ⅳ cases (*n* = 9). Although the model showed a high AUC for this category, the statistical reliability of this result is constrained by the small sample size. Future studies should employ larger, balanced multi-center cohorts to further validate the model’s generalizability in severe disease. Third, the 3D DenseNet was trained on CT images; by the time CT is used for diagnosing sacroiliitis, obvious structural bone damage has already occurred, meaning the optimal treatment window may have been missed. Fourth, the 3D DenseNet only provides a preliminary grading diagnosis of sacroiliitis. Misclassification occurs to some extent between adjacent categories (e.g., category 0 vs. 1, category 1 vs. 2), especially with grade Ⅰ sacroiliitis, which is prone to under-diagnosis. Future research should explore extended models that integrate multimodal data to further improve the model’s ability to correctly identify subtle bone erosions and enable earlier diagnosis of sacroiliitis.

## Conclusion

We developed and validated a 3D DenseNet—an integrated segmentation–classification 3D-CNN—for automated diagnosis of sacroiliitis. The model achieved high diagnostic accuracy for both detailed five-grade classification and a simplified three-class clinical task, and demonstrated robust stability and generalizability in internal and external validation cohorts. By reducing the subjectivity of manual grading, it provides reproducible assessments and supports accurate diagnosis, risk stratification, and disease monitoring of sacroiliitis.

## Data Availability

The datasets used and/or analyzed during the current study are available from the corresponding author on a reasonable request.
